# Cytotoxic diterpenoids from *Salvia glutinosa* and comparison with the tanshinone profile of danshen (*Salvia miltiorrhiza*)

**DOI:** 10.3389/fpls.2023.1269710

**Published:** 2023-12-04

**Authors:** Arpine Ayvazyan, Lenard Deutsch, Christian Zidorn, Brigitte Kircher, Serhat S. Çiçek

**Affiliations:** ^1^ Department of Pharmaceutical Biology, Kiel University, Kiel, Germany; ^2^ Tyrolean Cancer Research Institute, Innsbruck, Austria; ^3^ Immunobiology and Stem Cell Laboratory, Department of Internal Medicine V (Hematology and Oncology), Innsbruck Medical University, Innsbruck, Austria; ^4^ Department of Biotechnology, Hamburg University of Applied Sciences, Hamburg, Germany

**Keywords:** diterpene, tanshinone, cytotoxicity, *Salvia miltiorrhiza*, *Salvia glutinosa*, proliferation, danshen

## Abstract

The roots of *Salvia miltiorrhiza* are the source of the traditional Chinese medicine danshen and the class of tanshinones, particular quinoid *nor*-diterpenoids of the abietane type. Of these compounds, cryptotanshinone, dihydrotanshinone I, tanshinone I, and tanshinone IIA, have been extensively studied for their anticancer potential, not only but as well because of their high abundance in *S. miltiorrhiza* and their thus easy availability. However, also additional *Salvia* species are known to contain tanshinones, mainly such of the subgenus *Glutinaria*, of which *S. glutinosa* is the only species widely occurring in Europe. Using UHPLC-DAD-MS, the tanshinone profile of *S. glutinosa* roots collected from two different locations was compared to the profile in *S. miltiorrhiza* roots. In addition, tanshinone IIA and another six diterpenoids from *S. glutinosa* were investigated for their antiproliferative and cytotoxic potential against MDA-MB-231 and HL-60 cells. Apart from dihydrotanshinone I, which has been previously characterized due to its anticancer properties, we determined danshenol A as a highly antiproliferative and cytotoxic agent, significantly surpassing the effects of dihydrotanshinone I. With regard to the diterpenoid profile, *S. miltiorrhiza* showed a higher concentration for most of the tanshinones, except for (+)-danshexinkun A, which was present in comparable amounts in both species. Danshenol A, in contrast, was only present in *S. glutinosa* as were dehydroabietic acid and (+)-pisiferic acid. The results of our study underlines the long traditional use of danshen due to its high amount on tanshinones, but also demonstrates the potential value of investigating closely related species for the discovery of new biologically active lead compounds.

## 1 Introduction

*Salvia* is the largest genus of the Lamiaceae family, with over 900 species widely distributed throughout the world (Hu et al., 2018). The genus is known for its long-standing use in traditional medicine and its diterpene richness (Wu et al., 2012; Wei et al., 2017). One of the most important species is *Salvia miltiorrhiza* Bunge, which has been part of traditional Chinese medicine since ancient times (Chun-Yan et al., 2015). It has been officially recorded in the Chinese pharmacopoeia since 1963 (Guo et al., 2014) and is used for the treatment of various diseases, such as Alzheimer’s disease (Wong et al., 2010), diabetes (Jia et al., 2019), cerebrovascular disease (Zhou et al., 2011), coronary heart disease (Lam et al., 2006), cancer (Zhang and Wang, 2006), hepatocirrhosis (Lin et al., 2006), and Parkinson’s disease (Zhang et al., 2010). In Chinese traditional medicine there were two primary types of *S. miltiorrhiza* preparations used by patients: directly prepared herbal raw materials (decoctions, extracts, tinctures) and Chinese patent medicines (water pills, honey pills). However, the use of other products such as injection and ultrafine granular powder is increasing significantly (Shen et al., 2017). One of the preparations of modern Chinese medicine, namely compound Danshen dripping pills, which consist of *S. miltiorrhiza*, *Panax notoginseng*, and borneol, is now widely used in China for the prevention and treatment of angina pectoris, hyperlipidemia and coronary heart disease (Chu et al., 2011). In addition, danshen is used as a healing tea to boost immunity and prevent diseases (Wei et al., 2017).

*S. miltiorrhiza* has attracted much attention due to its content on tanshinones (Jiang et al., 2019), a class of phenolic abietane-type *nor*-diterpenoids possessing a 14,16-ether D-ring (Ma et al., 2021). Tanshinones were repeatedly isolated and investigated because of their bioactive properties such as anti-inflammatory (Ma et al., 2016), antitumor (Jiang et al., 2019), antibacterial and antiviral (Lai et al., 2021), and antioxidant activities (Li et al., 2013; Sun et al., 2014). Thereby, the cytotoxic activity of selected tanshinones, e.g., tanshinone I and IIA, cryptotanshinone, and dihydrotanshinone I, is of special interest. Tanshinone IIA has been shown to inhibit proliferation and to induce apoptosis of human hepatocellular carcinoma BEL-7402 and SMMC-7721 cells (Yuan et al., 2004) as well as human promyelocytic leukemic cells (HL-60) and human erythroleukemic cells (K562) (Sung et al., 1999; Yoon et al., 1999). It was, furthermore, reported that cryptotanshinone inhibits the growth of H446 lung tumor cells (Man et al., 2016), suppresses cell viability and induces apoptosis of the breast cancer cell line MCF-7 (Park et al., 2012), and induces apoptosis and inhibits movement of CEM/ADR5000 and CCRF-CEM lymphoblastic leukemia cells (Wu et al., 2016). In addition, tanshinone I and dihydrotanshinone I were found to induce apoptosis in human gastric cancer cell lines SGC-7901 and MGC-803 (Cheng et al., 2016; Jing et al., 2016) and breast cancer cell lines MDA-MB-231 and MCF-7 (Tsai et al., 2007; Nizamutdinova et al., 2008).

The compound class of tanshinones, however, is not exclusive to *S. miltiorrhiza*. There have been reports from more than twenty different species containing these particular compounds, such as *S. digitaloides, S. przewalskii*, or *S. yunnanensis*, with most of them occurring in East Asia (Zhang et al., 2012). In addition, *S. glutinosa*, a plant species widely distributed in Europe but with similar floral morphology to *S. miltiorrhiza* is known to contain tanshinone derivatives (Nagy et al., 1998; Hu et al., 2018). To date, many phylogenetic studies have been conducted that have divided *Salvia* into several clades or subgenera placing *S. miltiorrhiza* in the same clade or subgenus as *S. glutinosa* (Drew et al., 2017; Will and Claßen-Bockhoff, 2017; Hu et al., 2018; Kriebel et al., 2019; Rose et al., 2021; Wu et al., 2021; Moein et al., 2023). However, *S. glutinosa* differs from other species of the same subgenus or clade in that it is the only one distributed not only in Asia but also in Europe, while the other species are mainly represented by Chinese species (Will and Claßen-Bockhoff, 2017).

*S. glutinosa*, which is referred to as glutinous sage or Jupiter’s sage, is an aromatic perennial plant that grows in moist forests from Central and Eastern Europe to Western Asia (Kaya et al., 2003). There is plenty of evidence of the traditional use of glutinous sage in folk medicine in many countries. In Romania, a decoction of the roots was used against dizziness and an infusion of the flowers and leaves was administered against gastroenteritis and cough pains and to treat injuries and animal bites (Nicolescu et al., 2022). The leaves of *S. glutinosa* are also used to prepare an infusion for digestion and to treat headaches and sore throats (Idolo et al., 2010) or for culinary purposes as an aromatic herb (Motti, 2021).

After the first reports for tanshinones in *S. glutinosa* (Nagy et al., 1998), additional derivatives were isolated from this species, inter alia the well-known tanshinone I, tanshinone IIA, cryptotanshinone, or dihydrotanshinone I (Nagy et al., 1999; Senol et al., 2017; Ayvazyan et al., 2023). However, no reports on the content of tanshinones in *S. glutinosa* have been made so far. Because of the long maturation time of *S. miltiorrhiza* and growing pharmaceutical demand for tanshinones, alternative sources for this particular compound class are becoming of increased interest. Thus, the aim of our study was to determine the amount of the major tanshinones in *S. glutinosa* roots and to identify eventual cytotoxic constituents, which are less abundant in danshen and therefore not yet biologically characterized.

## 2 Materials and methods

### 2.1 Plant materials and chemical reagents

Roots of *S. glutinosa* (Sample numbers SAG_A and SAG_I) were collected in two different locations: SAG_A in Amlach, East Tyrol, Lienz/Tyrol, Austria (coordinates: N 46°48’34’’, E 12°46’00’’; alt.: 694 m; in June 2021). SAG_I: Sillschlucht, Innsbruck/Tyrol, Austria (coordinates: N 47°14’50’’, E 11°24’10’’; alt.: 640 m; August 2021). Plants were identified by Prof. Serhat Sezai Çiçek using the Austrian standard flora (Fisher et al., 2008) and vouchers were deposited in the Herbarium of Kiel University (KIEL0005018 and KIEL0005019, respectively). The roots of *S. miltiorrhiza* (source: Linyi, Shandong, Batch 03008) were purchased from China Medica, Weissach, Germany.

Acetonitrile and water (both of LC-MS grade), gradient grade methanol, gradient grade ethanol and other (analytical grade) solvents were obtained from VWR International GmbH, Darmstadt, Germany. The isolation of diterpenes 1 – 6 from *S. glutinosa* roots is described in Ayvazyan et al. (2023). An isolation tree is presented in **Supplementary Figure 1**. Tanshinone IIA (compound **7**) was obtained from Extrasynthese, Genay, France (ID: 0110/0) and (+)-vouacapenic acid was isolated as described in Çiçek et al. (2022). Purity of the compounds was determined with 92.7% and 95.4%, respectively using quantitative NMR and the ERETIC method (Electronic REference To access *In vivo* Concentrations). NMR spectra are depicted in **Supplementary Figures 2** and **3**.

### 2.2 Quantification of tanshinones by UHPLC

Sample preparation and quantification of eleven diterpenoids was carried out according to the method by Li et al. (2008) with slight modifications: A Kinetex C18 column (100 x 2.1 mm, 1.7 µm particle size, Phenomenex, Aschaffenburg, Germany) was used and the gradient was adjusted to 0-5 min 42-45% B, 5-11 min 45-56% B, 11-16 min 56-85% B, and 16-20 min 85-95% B. Quantitative analysis and confirmation of peaks by UHPLC-PDA and LC-MS, respectively, were done on an Ultimate 3000 instrument equipped with an HPG-3400SD pump, a WPS-3000SL autosampler, a TCC-3000SD column heater, and a VWD-3400RS variable wavelength detector (Thermo Fisher Scientific Inc., Waltham, MA, USA) and a Nexera X2 instrument consisting of an LC-30AD binary pump, a SIL-30AC autosampler, a CTO-20AC column oven, and a SPD-M30A diode-array detector (Shimadzu, Kyoto, Japan). All samples were injected in triplicates. UHPLC-PDA chromatograms of reference standards and details of calibration as well as chromatograms of extracts and MS spectra of the quantified compounds are provided in the supporting information (**Supplementary Figures 4**-**17**).

### 2.3 General cell-culture methods

The mammary carcinoma cell line MDA-MB-231 and the acute myeloid leukemia cell line HL-60 were purchased from DSMZ – German Collection of Microorganisms and Cell Cultures, Braunschweig, Germany. The human stroma cell line HS-5 was kindly provided by the Tyrolean Cancer Research Institute. Cell-culture medium for all three cell lines consisted of RPMI 1640 without phenol red (PanBiotech, Aidenbach, Germany) supplemented with fetal bovine serum (10% FBS, Biowest, Nuaillé, France), L-Glutamine (2 mM), Penicillin (100 U ml^-1^) and Streptomycin (100 µg ml^-1^) (all from Sigma-Aldrich, Vienna, Austria). Cells were grown at 37°C under a 5% CO_2/_95% air atmosphere and were passaged twice weekly. The compounds were dissolved in DMSO (stock solution 50 mM). The final test concentration was reached upon dilution with RPMI 1640 without FBS.

### 2.4 Analysis of proliferation

Logarithmically growing MDA-MB-231 cells were seeded in triplicates in flat-bottomed 96-well plates with a density of 1 x 10^4^ cells in 50 µl per well. Logarithmically growing HL-60 cells were seeded in triplicates in U-bottomed 96-well plates with a density of 5 x 10^4^ cells in 50 µl per well. After overnight incubation (MDA-MB-231) or a two-hours incubation (HL-60) at 37°C under a 5% CO_2/_95% air atmosphere, the compounds were added to achieve the test concentrations at a final volume of 150 µl, respectively. During the last 12-16 h of incubation, each well was exposed to [^3^H]-thymidine (2 Ci mmol^-1^, Hartmann Analytic, Braunschweig, Germany). Cells were harvested after a total incubation time of 72 h by a semiautomated device and [^3^H]-thymidine uptake into cells expressed as counts per minute (cpm) was measured in a scintillation counter (Microbeta Trilux, Perkin Elmer, Waltham, MA, USA). Proliferation without complexes set at 100% served as negative control and the compounds' activity was calculated as percentage of the control.

### 2.5 Analysis of metabolic activity

The logarithmically growing adherent cell lines MDA-MB-231 and HS-5, as well as HL-60 cells were seeded and treated as described above. After 72 h, the cells were examined for their metabolic activity using a modified MTT assay (EZ4U kit; Biomedica, Vienna, Austria) according to the manufacturer´s protocol. Unspecific staining generated by the FBS-containing medium was excluded by subtracting the optical density of the cell culture medium. Metabolic activity in the absence of the compounds was set at 100%.

### 2.6 Scratch assay/wound healing assay

Logarithmically growing MDA-MB-231 cells (1 x 10^6^) were resuspended in 4 ml RPMI 1640 medium supplemented with 10% FBS, plated in 12-well plates (Greiner bio-one, Kremsmünster, Austria) and incubated at 37°C in 5% CO_2/_95% air for 24 h. Thereafter, a scratch was performed on the confluent cell layer with a 200 µL pipette tip. The compounds were added immediately at a concentration of 10 µM and the cells were further incubated for 72 h at 37°C in a 5% CO_2/_95% air atmosphere. Pictures were taken with a life-cell imaging microscope (Juli Smart Fluorescent Cell Analyser, Digital Bio/NanoENTek, Seoul, South Korea) every 24 h to document the closing of the scratch.

### 2.7 Statistical analysis

The Mann Whitney U Test was used to analyze the differences between proliferation and metabolic activity in the absence and the presence of a variable concentration of the test compounds (NCSS software, Kaysville, UT, USA). A p value <0.05 represents statistical significance.

## 3 Results

### 3.1 Quantification of tanshinone diterpenes by UHPLC

**Figure 1:**
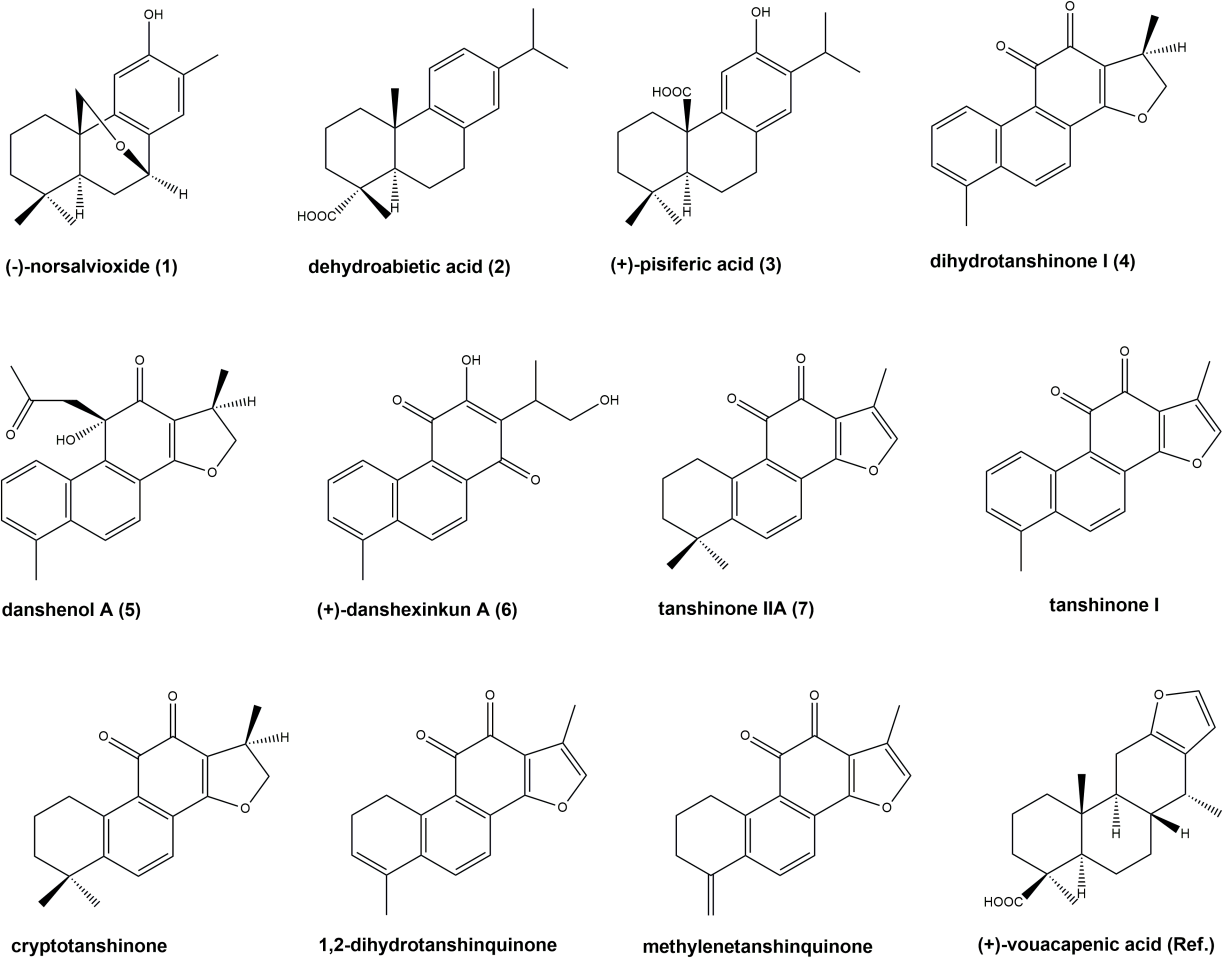
Chemical structures of quantified compounds: (–)-norsalvioxide (1), (+)-dehydroabietic acid (2), (+)-pisiferic acid (3), dihydrotanshinone I (4), danshenol A (5), (+)-danshexinkun A (6), tanshinone IIA (7), tanshinone I, cryptotanshinone, 1,2-dihydrotanshinquinone, and methylenetanshinquinone, as well as reference standard (+)-vouacapenic acid. Numbered compounds were additionally subjected to biological assays.

**Figure 2:**
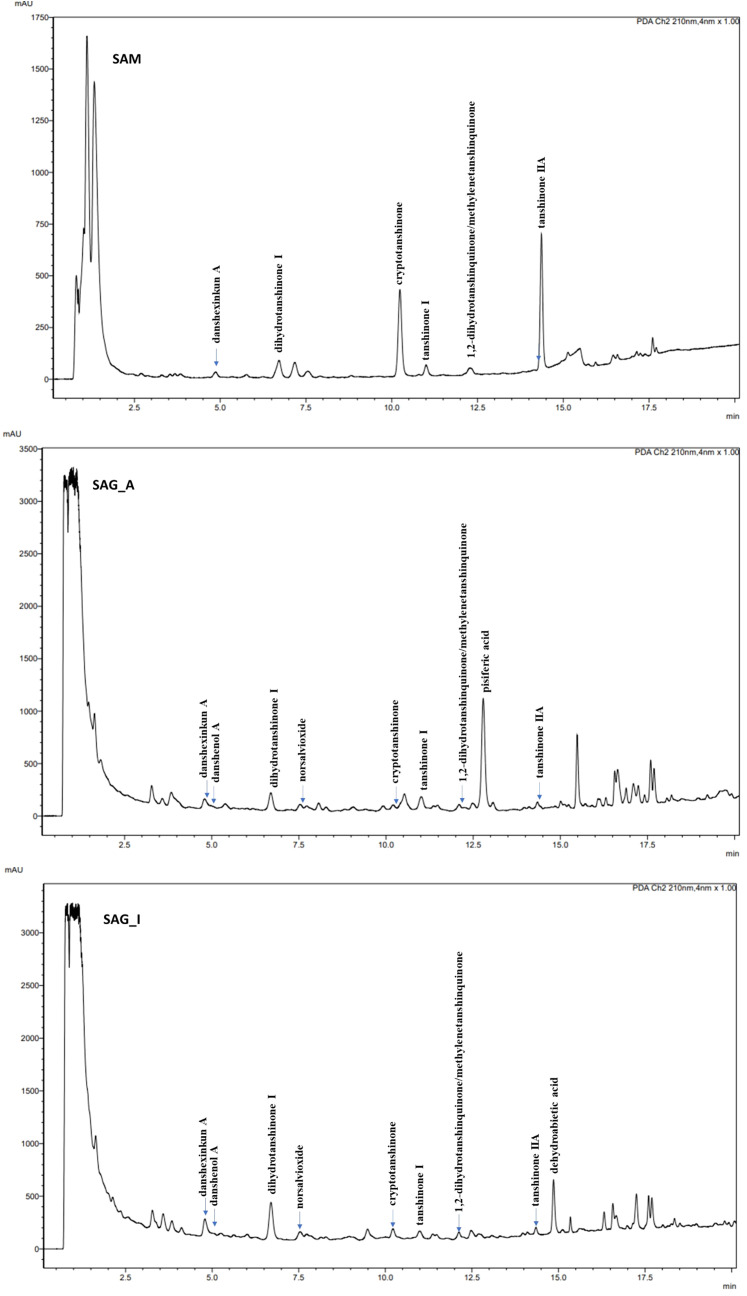
UHPLC-PDA chromatograms of *Salvia miltiorrhiza* (SAM, top), *Salvia glutinosa* from Amlach (SAG_A, middle), and *Salvia glutinosa* from Innsbruck (SAG_I, bottom) ethanol extract at a wavelength of 210 nm. Column: Kinetex C18 (100 x 2.1 mm, 1.7 µm particle size). Solvent system: water (A) and acetonitrile (B). Gradient: 0 min (42% B), 5 min (45% B), 11 min (56% B), 16 min (85% B), 20 min (95% B). Column temperature: 25°C. Flow rate: 0.3 mL/min. Injection: 1 µL. Compounds: danshexinkun A (6), danshenol A (5), dihydrotanshinone I (4), norsalvioxide (1), cryptotanshinone, tanshinone I, 1,2-dihydrotanshinquinone/methylenetanshinquinone, pisiferic acid (3), tanshinone IIA (7), and dehydroabietic acid (2).

*S. glutinosa* root samples from two different locations were investigated for their content on eleven diterpenoids, namely (–)-norsalvioxide (1), (+)-dehydroabietic acid (2), (+)-pisiferic acid (3), dihydrotanshinone I (4), danshenol A (5), and (+)-danshexinkun A (6), tanshinone IIA (7), tanshinone I, cryptotanshinone, 1,2-dihydrotanshinquinone, and methylenetanshinquinone, whereby the latter two compounds were quantified together (**Figures 1**, **2**). The method used for quantitation was developed by Li et al. (2008) for the analysis of *S. miltiorrhiza*, which was also quantified in our study (**Figure 2**). (+)-Vouacapenic acid was used as calibrant for the quantification of compounds 1 to 3, while tanshinone IIA (7) was used to quantify the remaining (tanshinone-type) diterpenoids. The results are given in **Table 1** and show the amounts given in mg/kg dried plant material. In *S. miltiorrhiza*, tanshinone IIA (7) and cryptotanshinone were the most abundant of the quantified compounds, with amounts of 895 and 764 mg/kg, respectively. The concentrations of (–)-norsalvioxid (1), tanshinone I, dihydrotanshinone I (4), and 1,2-dihydrotanshinquinone/methylenetanshinquinone, however, were ten-fold lower, with amounts ranging from 72 to 88 mg/kg. Dehydroabietic acid (2), (+)-pisiferic acid (1), and danshenol A (5) were not detected in *S. miltiorrhiza*, whereas (+)-danshexinkun A (6) was determined with 52 mg/kg dried plant material. *S. glutinosa*, in contrast, displayed a much lower content on the major danshen tanshinones, with amounts ranging from 3.4 mg/kg to 10.9 mg/kg in the samples collected from Amlach (SAG_A) and amounts ranging from 1.6 to 15.7 mg/kg in the samples collected from Innsbruck (SAG_I) (**Table 1**). However, both samples showed slightly higher levels of (+)-danshexinkun A (6), being the major diterpenoid in SAG_I, as well as the presence of danshenol A (5), which was not found in *S. miltiorrhiza*. The two *S. glutinosa* samples mostly differed between each other in the presence of dehydroabietic acid (2) and (+)-pisiferic acid (3), respectively. While dehydroabietic acid (2) was only detected in the sample collected from Innsbruck (SAG_I), (+)-pisiferic acid (3) was only found in the sample collected from Amlach (SAG_A), thereby even being the major diterpenoid with an amount of 112 mg/kg.

**Table 1:** Content of diterpenes in *Salvia miltiorrhiza* (SAM) as well as *Salvia glutinosa* collected from Amlach (SAG_A) and Innsbruck (SAG_I).

Diterpenes	SAM	SAG_A	SAG_I
(–)-norsalvioxide (**1**)	87.6 ± 7.9	8.7 ± 0.8	12.4 ± 0.8
dehydroabietic acid (**2**)	n.d.	n.d.	18.6 ± 0.1
(+)-pisiferic acid (**3**)	n.d.	112.0 ± 0.2	n.d.
dihydrotanshinone I (**4**)	75.8 ± 4.0	6.9 ± 1.2	15.7 ± 1.1
danshenol A (**5**)	n.d.	8.6 ± 0.2	5.6 ± 0.8
(+)-danshexinkun A (**6**)	51.8 ± 5.7	55.3 ± 0.8	57.4 ± 0.4
tanshinone IIA (**7**)	895.2 ± 94.2	3.4 ± 0.2	5.8 ± 0.5
cryptotanshinone	764.1 ± 70.7	6.6 ± 1.0	8.3 ± 0.8
tanshinone I	87.9 ± 4.2	10.9 ± 0.3	6.3 ± 0.5
1,2-dihydrotanshinquinone/methylenetanshinquinone	72.2 ± 2.2	4.1 ± 0.8	1.6 ± 0.2

Amounts are given in mg/kg dried plant material. n.d. means not detected.

### 3.2 Biological evaluation

Six isolated diterpenoids from *S. glutinosa* (1-6) as well as commercially obtained tanshinone IIA (7) were investigated for their antiproliferative and cytotoxic properties against the mammary carcinoma cell line MDA-MB-231 and the acute myeloid leukemia (AML) cell line HL-60. The chemical structures of the seven tested compounds are depicted in **Figure 1**. The non-malignant stroma cell line HS-5 was used as control to examine general cytotoxicity. Effectivity of the compounds was analyzed by inhibition of proliferation and metabolic activity in the range from 1 µM to 50 µM as well as by a scratch/wound healing assay.

#### 3.2.1 Antiproliferative activity

**Figure 3:**
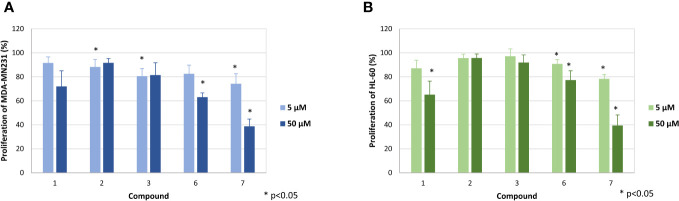
Effect of the compounds 1, 2, 3, 6 and 7 on the proliferation of MDA-MB-231 **(A)** and HL-60 cells **(B)**. The mean proliferation + standard deviation of four (MDA-MB-231) and five (HL-60) independent experiments is depicted. The proliferation of cells only (without compound) was set at 100% (data not shown). Asterisk (*) represents statistical significance against the cells without compound.

In a first step, the seven diterpenoids were evaluated for their antiproliferative activity, whereby the effect on the proliferation of MDA-MB-231 cells was determined by [^3^H]-thymidine uptake. Compounds 1-3 did not strongly diminish the proliferation of the MDA-MB-231 cells up to the highest concentration tested (**Figure 3A**), while compounds 6 and 7 inhibited the proliferation at 50 µM to 63.1 ± 3.5% and 38.7 ± 6.1%, respectively. Testing against HL-60 cells showed similar activities (**Figure 3B**), with only weak to no effects for compounds 2, 3, and 6 and moderate effects at the highest concentration for compounds 1 and 7, which inhibited proliferation to 65.1 ± 11.3% and 38.7 ± 8.8%, respectively.

**Figure 4:**
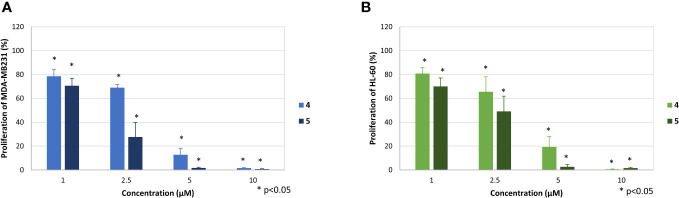
Effect of the compounds 4 and 5 on the proliferation of MDA-MB-231 **(A)** and HL-60 cells **(B)**. The mean proliferation + standard deviation of four (MDA-MB-231) and five (HL-60) independent experiments is depicted. The proliferation of cells only (without compound) was set at 100% (data not shown). Asterisk (*) represents statistical significance against the cells without compound.

Much stronger effects were observed for compounds 4 and 5, which dose-dependently reduced the proliferation of both cell lines already at 2.5 µM (**Figure 4**). At this concentration compound 4 reduced proliferation of MDA-MB-231 cells to 69.9 ± 2.6% and HL-60 cells to 65.5% ± 12.6%, respectively. At the same concentration compound 5 reduced proliferation of MDA-MB-231 cells to 27.6 ± 12.2 and of HL-60 to 49.2 ± 12.8%. Accordingly, proliferation of both cells lines was completely blocked at concentrations of 10 µM (4) and 5 µM (5), respectively.

#### 3.2.2 Metabolic activity

**Figure 5:**
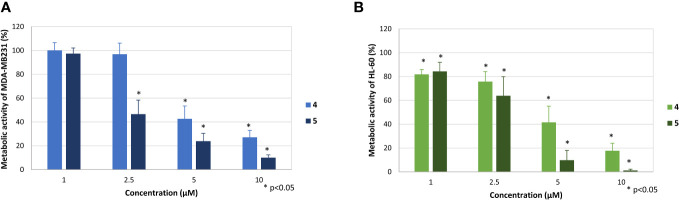
Effect of the compounds 4 and 5 on the metabolic activity of MDA-MB-231 **(A)** and HL-60 cells **(B)**. The mean metabolic activity + standard deviation of four (MDA-MB-231) and six (HL-60) independent experiments is depicted. The metabolic activity of cells only (without compound) was set at 100% (data not shown). Asterisk (*) represents statistical significance against the cells without compound.

Cytostatic activity may be accompanied by cytotoxicity. Therefore, the most active compounds 4 and 5 were evaluated by a modified MTT (3-(4,5-dimethylthiazol-2-yl)-2,5-diphenyl-tetrazolium bromide) test (**Figure 5**). This assay detects the reduction of light-yellow tetrazolium salts into orange colored formazan derivates, a reaction which can only be detected in the presence of functional mitochondria.

Both compounds dose-dependently inhibited the metabolic activity, whereby the effectivity was slightly weaker in comparison to the proliferation. The mitochondrial activity of MDA-MB-231 cells was reduced to 27.2 ± 5.7% (4) and 10.1 ± 2.3% (5) at a concentration of 10 µM and to 42.6% ± 10.9% (4) and 23.8 ± 6.5% (5), respectively (**Figure 5A**). Compound 5 still showed pronounced effects at a concentration of 2.5 µM, reducing metabolic activity of MDA-MB-231 to 46.6 ± 11.8%.

The effect was even stronger against HL-60 cells (**Figure 5B**). Here, significant reduction of mitochondrial activity was observed at all tested concentrations, especially at concentrations of 10 and 5 µM, respectively. At these concentrations, metabolic activity was reduced to 17.7% ± 6.3% and 41.5 ± 13.6% by compound 4 and to 9.8% ± 8.2% and 1.2 ± 1.2% by compound 5. Also, at a concentration of 1 µM slight but significant reductions to 81.8% ± 4.0% (4) and 84.1 ± 7.6% (5) were observed.

**Figure 6:**
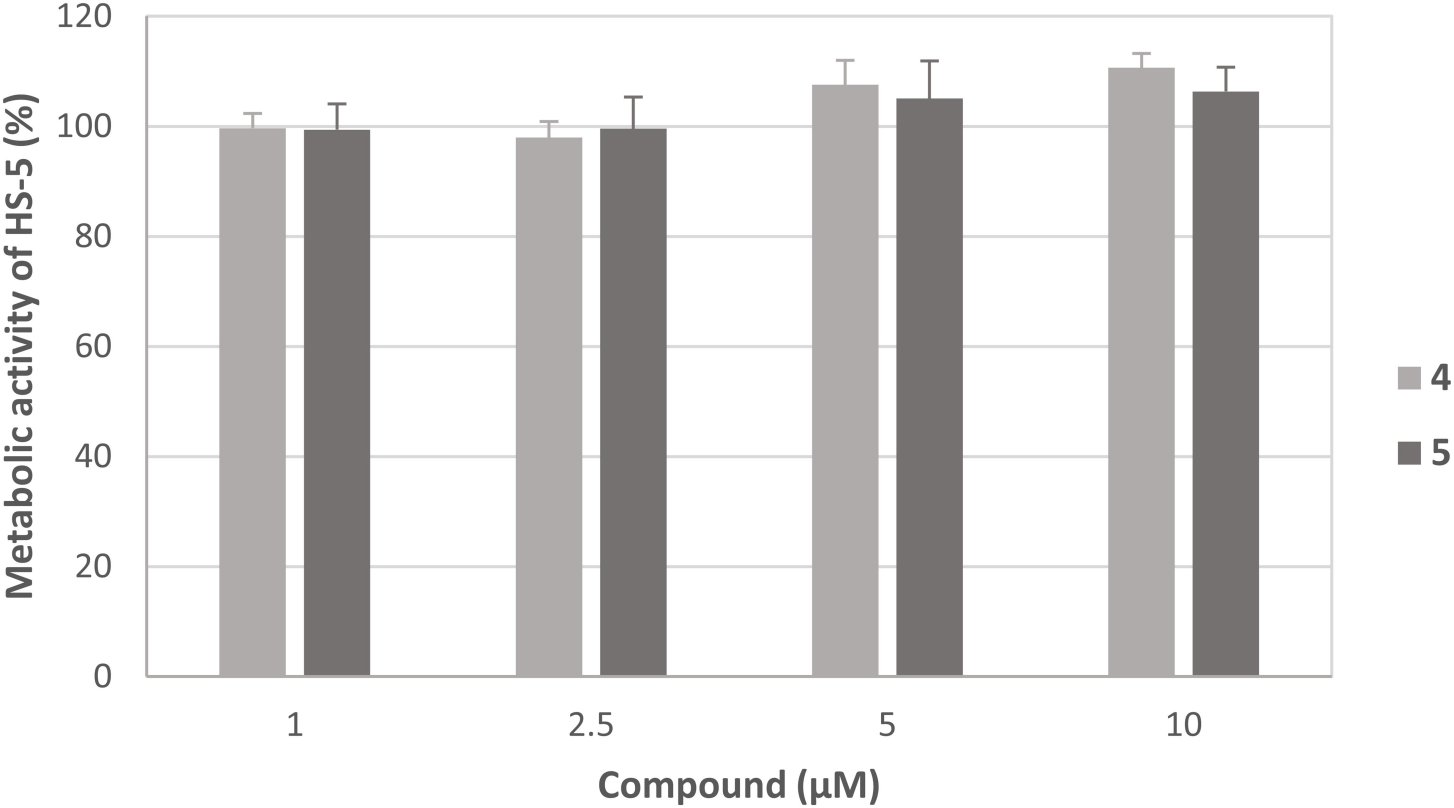
Effect of compounds 4 and 5 on the metabolic activity of HS-5 cells. The mean metabolic activity + standard deviation of four independent experiments is depicted. The metabolic activity of cells only (without compound) was set at 100% (data not shown).

In order to evaluate whether the observed effects were caused by general cytotoxic activity, both compounds were analyzed on the non-malignant human stroma cell line HS-5. Interestingly, the metabolic activity was not significantly influenced by both compounds suggesting tumor-selectivity of compounds 4 and 5 (**Figure 6**).

#### 3.2.3 Wound healing

**Figure 7:**
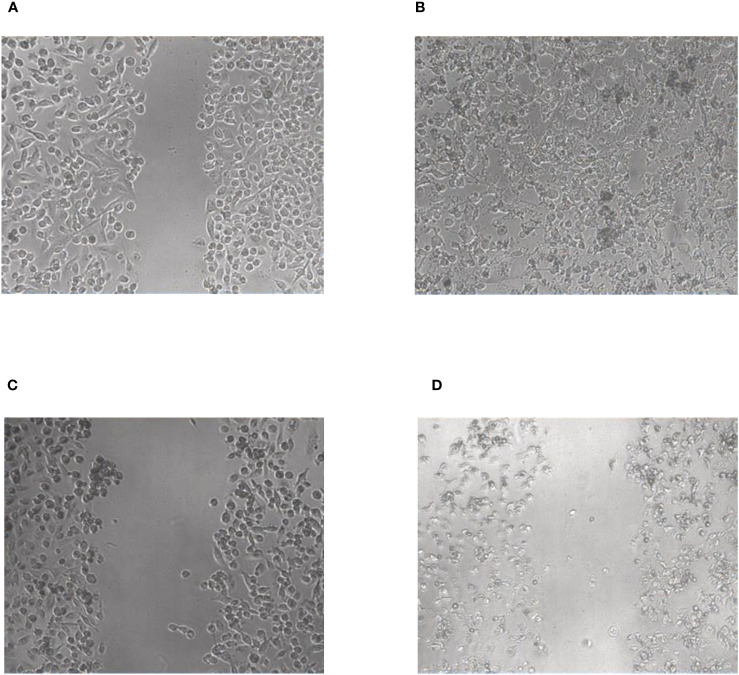
Wound healing/scratch assay with compound 2 **(A, B)** and compound 5 **(C, D)**, respectively. The pictures were taken immediately after performing the scratch **(A, C)** and after 72 h **(B, D)**.

To further investigate the inhibitory potential of the compounds, a wound healing/scratch assay was performed (**Figure 7**). In this assay the confluent cell layer of MDA-MB-231 cells was scratched with a 200 µl pipette tip and the migratory potential of the compounds to close this scratch was analyzed microscopically by taking pictures immediately after compound addition and after 72 hours. Despite addition of 10 µM of compound the scratch was completely closed and seemed to be even overgrown by the cells after 72 hours of incubation (**Figures 7A, B**). In contrast, the cells treated with 10 µM of compound 5 completely lost their epithelial morphology and displayed the feature of dead cells which obviously were not able to close the scratch anymore (**Figures 7C, D**), thus confirming the results of the proliferation and metabolic activity assays.

## 4 Discussion

Diterpenoids of the genus *Salvia* and in particular the group of tanshinones have been extensively studied for their pharmacological properties in the last decades. Especially their potential use in cancer therapy has been of interest, as several tanshinones were reported to exhibit pronounced apoptotic and cytotoxic effects. Tanshinone IIA (7), the major tanshinone in *S. miltiorrhiza* induced apoptosis in human leukemia cells by activation of caspase-3 (Sung et al., 1999). These effects were observed in K562 erythroleukemic cells and HL-60 promyelocytic cells, which were also investigated in our study. The compound, furthermore, inhibited growth and proliferation of human hepatocellular carcinoma cells by apoptosis induction (Yuan et al., 2004) and showed additive effects with taxol against Tau protein-related resistance in MCF-7 cells (Lin et al., 2017). Cryptotanshinone, the second major tanshinone in the *S. miltiorrhiza* samples of our study, was also studied in MCF-7 cells, thereby showing antiproliferative and apoptotic effects via the PI3K/AKT signalling pathway (Shi et al., 2022). The compound, moreover, affected leukemia cells, namely acute lymphoblastic leukemia cells, via inhibition of NFκB (Wu et al., 2016). In addition, cryptotanshinone showed apoptotic and antiproliferative effects against hepatic stellate cells (Hou et al., 2023) and inhibited lung tumor growth via activation of the JAK2/STAT4 pathway and increased CD4^+^ T cell toxicity (Man et al., 2016). In another study on lung cancer cells, which also included tanshinone IIA (7) and cryptotanshinone, tanshinone I was found to inhibit proliferation of the human lung adenocarcinoma cell line CL1-5 by 73% at a concentration of 10 µg/ml after 48 hours of incubation, whereas the other two tanshinones showed an inhibition of 50% (Lee et al., 2008). Subsequent *in vivo* studies demonstrated that the compound also reduced tumorigenesis and metastasis in mice. Here, the aromatic A-ring of tanshinone I (and a thus missing methyl group at position C-4) must be the reason for the stronger effects compared to tanshinone IIA (7) and cryptotanshinone, which both show an aliphatic A-ring and two methyl groups instead. Tanshinone I was, furthermore, found to induce apoptosis in gastric cancer cells (Jing et al., 2016) as well as in MCF-7 and MDA-MB-231 breast cancer cells (Nizamutdinova et al., 2008). The same two breast cancer cell lines were also used in a study by Tsai et al. (2007), who compared 15,16-dihydrotanshinone I (4), cryptotanshinone, and tanshinone I for their cytotoxic activity. After dihydrotanshinone I (4) showed the strongest effects, further studies found the compound to inhibit cancer cell proliferation and tumor growth in MDA-MB-231 cells. Same as tanshinone I, dihydrotanshinone I (4) bears an aromatic A-ring but also a dihydrofuran D-ring, which seems to be responsible for the increased activity compared to tanshinone I (bearing a furan D-ring instead). The stronger cytotoxic activity of dihydrotanshinone I (4) compared to other tanshinones was also emphasized by Cheng et al. (2016), who reported the compound to induce growth inhibition and apoptosis in gastric cancer cells.

The stronger antiproliferative effects of dihydrotanshinone I (4) compared to e.g., tanshinone IIA (**7**) was also corroborated by the results our study. While tanshinone IIA (7) reduced the proliferation of both MDA-MB-231 and HL-60 cells to 80% at a concentration of 5 µM (**Figure 2**), dihydrotanshinone I (4) exhibited the same effect at a concentration of 1 µM (**Figure 3**). At 5 µM instead, dihydrotanshinone I (4) reduced proliferation to less than 20% (**Figure 3**) and metabolic activity of both cell lines to approximately 40% (**Figure 4**), thus demonstrating the abovementioned cytotoxic potential. Another fact that was evident by our results is the lower proliferative activity of non-tanshinone abietane type diterpenoids, such as compounds 1 to 3 (**Figure 2**). Also, the only *p*-quinoid tanshinone (compound 6), did not exhibit noteworthy effects. However, dihydrotanshinone I (4) was not the most active compound observed in our study, but the structurally closely related danshenol A (5, **Figure 1**). Same as dihydrotanshinone I (4), danshenol A (5) bears an aromatic A-ring and a dihydrofuran D-ring and thus two of the features relevant for the pronounced effects in some of the aforementioned studies. However, in contrast to the keto function in position 11, which appear in the four major tanshinones (tanshinone IIA (7), cryptotanshinone, tanshinone I, and dihydrotanshinone I (4)), danshenol A (5) is substituted with an acetonyl group and a hydroxy group. Not possessing an *o*-quinone structure anymore, danshenol A (5) thus is to be regarded as a tanshinone derivative rather than a real tanshinone. However, with regard to its biological effects the compound surpassed the tanshinones (*o*- and *p*-quinones) and other diterpenoids investigated in our study including the highly active dihydrotanshinone I (4, **Figures 4**, **5**). Thereby, the effect on MDA-MB-231 cells are certainly of great interest, as both tanshinone I and dihydrotanshinone I (4) were reported to induce pronounced antiproliferative and apoptotic effects, with the latter compound also demonstrating anti-tumor potential *in vivo*. The antiproliferative effects against MDA-MB-231 cells in our study revealed a complete inhibition of proliferation at a concentration of 5 µM (which was achieved at 10 µM of compound 4) and a reduction to around 28% at 2.5 µM. At the same concentration dihydrotanshinone I (4) reduced proliferation to 70% and thus to a much lesser extent (**Figure 4A**). A similar picture was observed in the cytotoxicity assays (**Figure 5A**), where danshenol A (5) was approximately twice as active as dihydrotanshinone I (4) in the investigations on the HL-60 cell line (**Figures 4B**, **5B**). The strong cytotoxic potential of danshenol A (5), which was also evident in the wound healing assay (**Figure 7**), together with the missing toxicity against healthy cells (**Figure 6**) renders the compound a more than interesting compound for future studies. Even more so, as structurally related but probably less cytotoxic diterpenoids were repeatedly investigated and proven to exhibit various anticancer activities.

Of course, the amount of tanshinones found in *S. miltiorrhiza* is by far exceeding the concentrations in *S. glutinosa* (**Table 1**), which is an explanation for its long use in traditional medicine in contrast to the rather scarce mentions of the widely occurring glutinous sage. However, it also highlights a problem in modern phytopharmacology, where not seldom studies are accomplished with a few readily available compounds. This is most obvious for the tanshinones, where the majority of investigations were conducted with tanshinone IIA (7), cryptotanshinone, tanshinone I, and dihydrotanshinone I (4), thereby omitting eventual more promising candidates. Danshenol A (5) could be such a candidate, which became of interest in the last years due to its positive anti-atherosclerotic and anti-inflammatory effects (Zhao et al., 2017). Though the compound was first isolated from *S. miltiorrhiza*, it is not among the principal components in danshen and was also not detected in our study. Therefore, it was probably not considered for pharmacological studies. In contrast, it was one of the first two compounds isolated from *S. glutinosa* (Nagy et al., 1998) and was found in both of our collections (**Table 1**). Thus, phytochemical studies on taxonomically related species of well-known medicinal plants are a valuable strategy to discover potent bioactives, even though the investigated species itself is of only limited medicinal interest.

In conclusion, the present study demonstrated promising antiproliferative and cytotoxic effects for danshenol A (5) isolated from the glutinous sage (*Salvia glutinosa*). Though our analysis of *S. glutinosa* did not reveal the species as a potent source for tanshinones in terms of quantity, its particular tanshinone derivative danshenol A (5) was surpassing the effects of dihydrotanshinone I (4), which itself is regarded as one of the most cytotoxic tanshinones. Hence, further studies on the anticancer properties of danshenol A (5) are certainly warranted.

## Data availability statement

The original contributions presented in the study are included in the article/**Supplementary Material**. Further inquiries can be directed to the corresponding author.

## Ethics statement

Ethical approval was not required for the studies on humans in accordance with the local legislation and institutional requirements because only commercially available established cell lines were used.

## Author contributions

AA: Data curation, Investigation, Writing – original draft. LD: Data curation, Investigation, Writing – original draft. CZ: Writing – original draft, Resources. BK: Supervision, Writing – original draft, Data curation, Investigation. SÇ: Conceptualization, Funding acquisition, Writing – original draft, Writing – review & editing.
