# Supplementary material for: Cytotoxic diterpenoids from Salvia glutinosa and comparison with the tanshinone profile of danshen (Salvia miltiorrhiza)
Source: Front Plant Sci. 2023 Dec 4;14:1269710. doi: 10.3389/fpls.2023.1269710 (PMC10729661; doi:10.3389/fpls.2023.1269710)
Supplement: Supplementary file 1 [file DataSheet_1.docx]

Supplementary Material

**Cytotoxic diterpenoids from *Salvia glutinosa* and comparison with the tanshinone profile of danshen (*Salvia miltiorrhiza*)**

**Arpine Ayvazyan, Lenard Deutsch, Christian Zidorn, Brigitte Kircher, Serhat S. Çiçek***

*** Correspondence:** Serhat S. Çiçek: [Serhat](mailto:Serhat)Sezai.Cicek@haw-hamburg.de

# Supplementary Figures


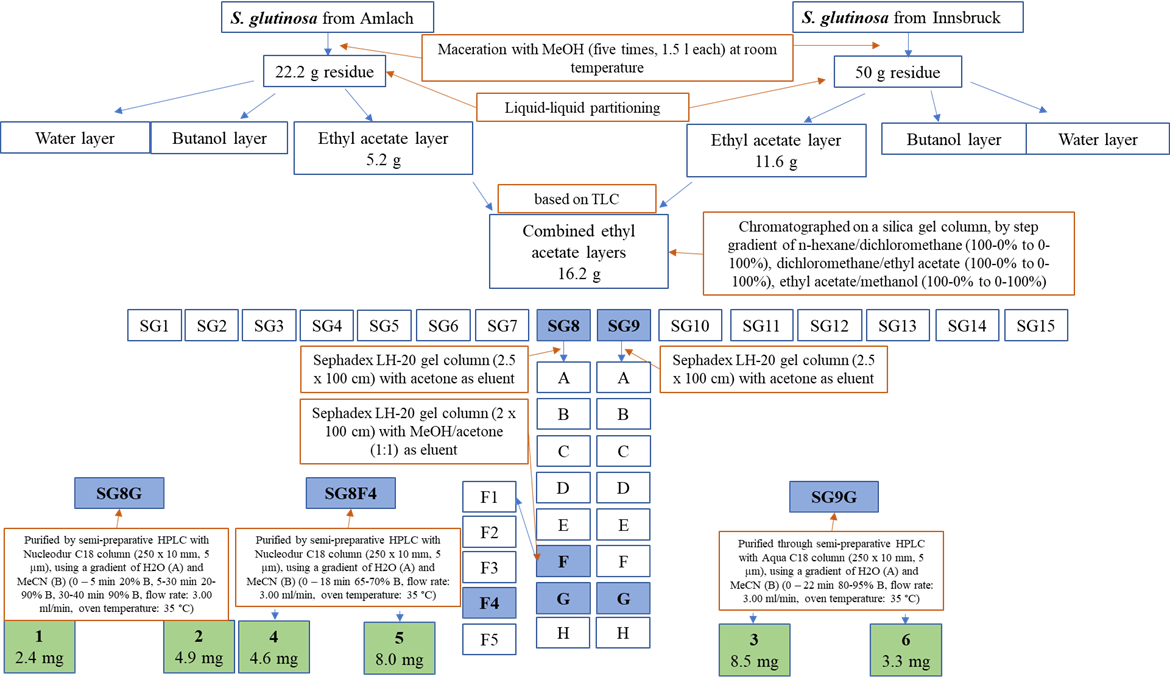


**Supplementary Figure 1.** Isolation of compounds **1**–**6** from the roots of *S. glutinosa*.


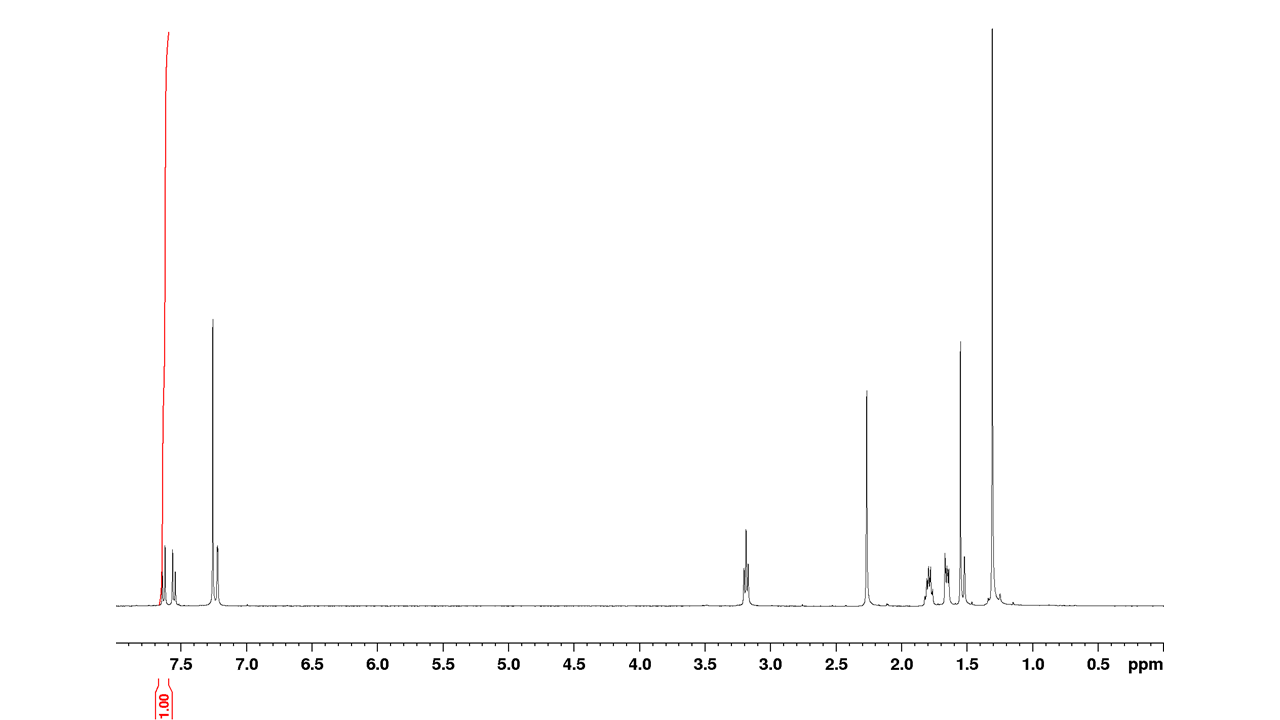


**Supplementary Figure 2**. ^1^H NMR spectrum of tanshinone IIA (**7**) measured in chloroform-*d*.


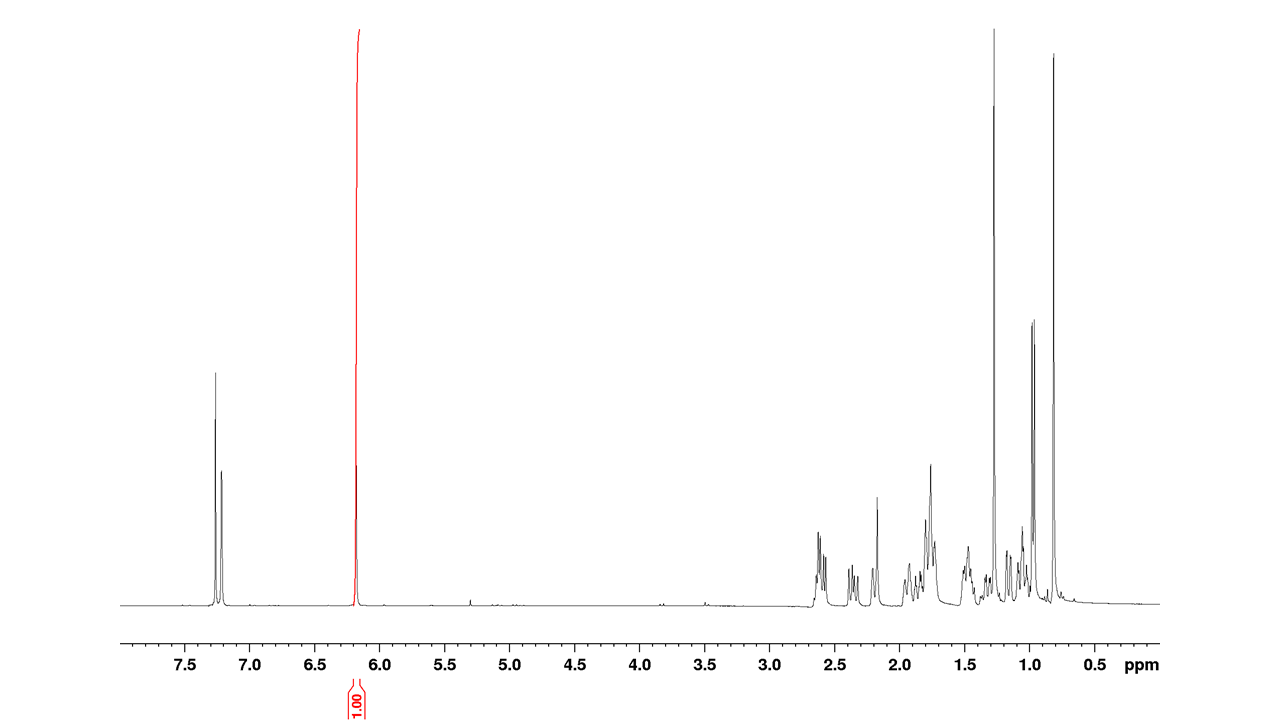


**Supplementary Figure 3**. ^1^H NMR spectrum of vouacapenic acid measured in chloroform-*d*.


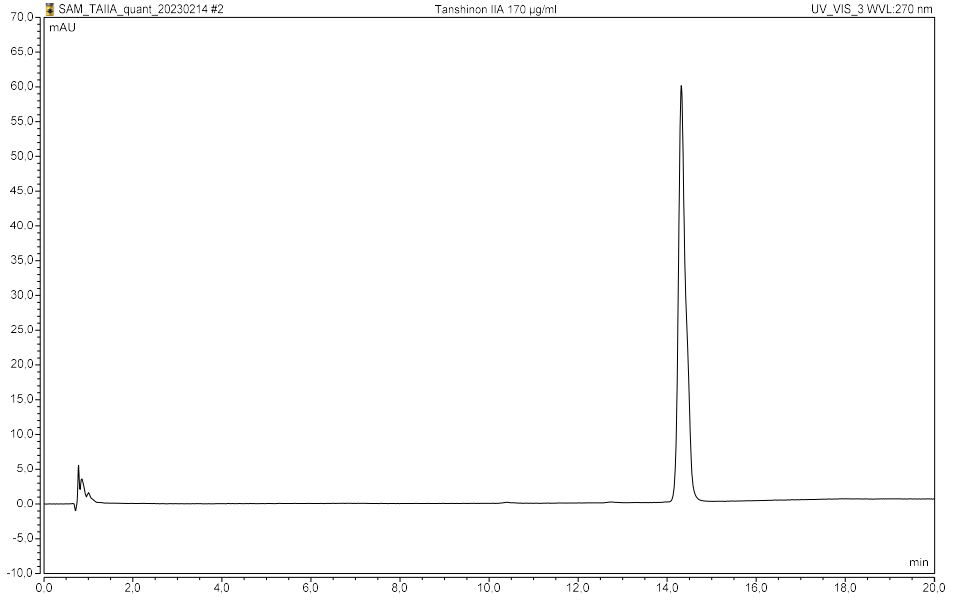


**Supplementary Figure 4**. UHPLC-PDA chromatogram of tanshinone IIA (**7**) at a wavelength of 270 nm. Column: Kinetex C18 (100 x 2.1 mm, 1.7 µm particle size). Solvent system: water (A) and acetonitrile (B). Gradient: 0 min (42% B), 5 min (45% B), 11 min (56% B), 16 min (85% B), 20 min (95% B). Column temperature: 25°C. Flow rate: 0.3 mL/min. Injection: 1 µL.


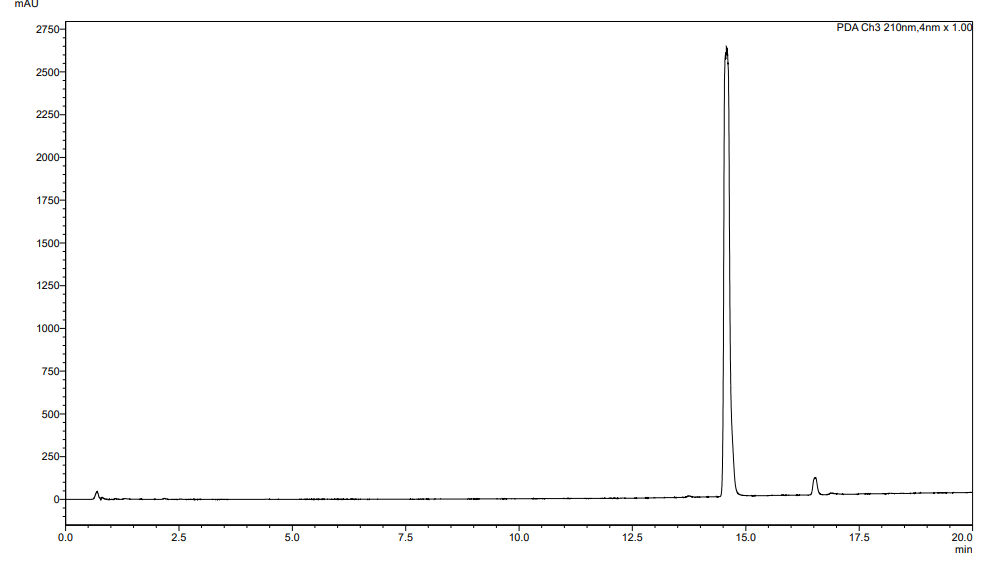


**Supplementary Figure 5**. UHPLC-PDA chromatogram of voucapenic acid at a wavelength of 210 nm. Column: Kinetex C18 (100 x 2.1 mm, 1.7 µm particle size). Solvent system: water (A) and acetonitrile (B). Gradient: 0 min (42% B), 5 min (45% B), 11 min (56% B), 16 min (85% B), 20 min (95% B). Column temperature: 25°C. Flow rate: 0.3 mL/min. Injection: 1 µL.

**Supplementary Figure 6.** Details on calibration curve for tanshinone IIA (**7**).

**Supplementary Figure 7.** Details on calibration curve for voucapenic acid.


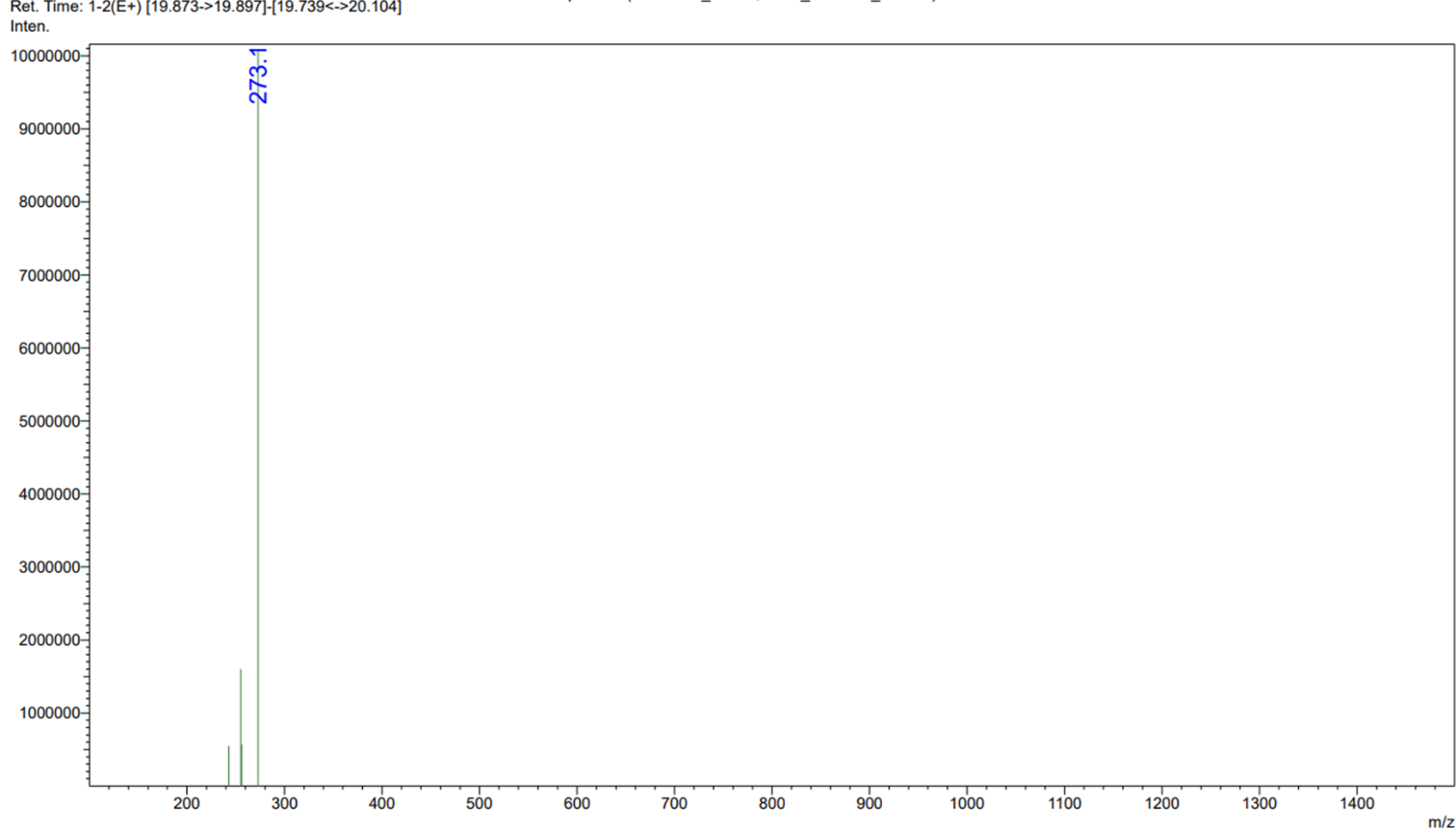
**Supplementary Figure 8.** On-line mass spectrum of (–)-norsalvioxide (**1**).


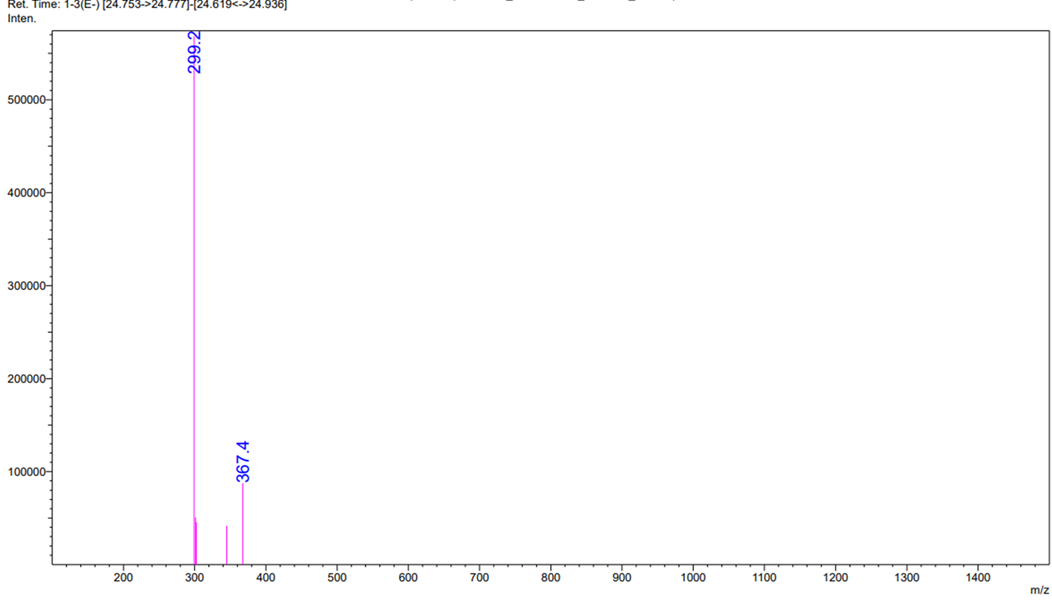
**Supplementary Figure 9.** On-line mass spectrum of dehydroabietic acid (**2**).

***
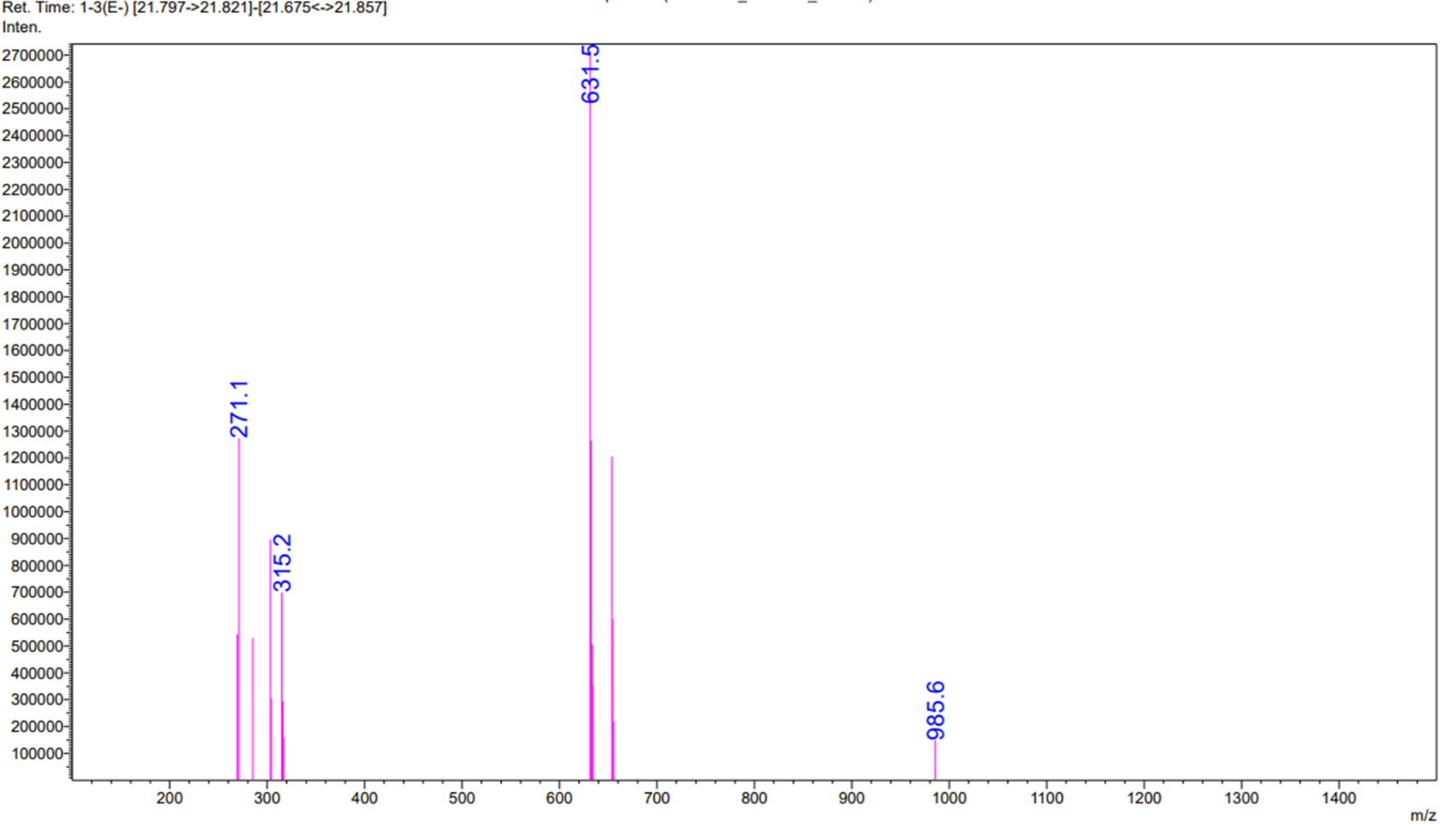
*Supplementary Figure 10.** On-line mass spectrum of (+)-pisiferic acid (**3**).

**
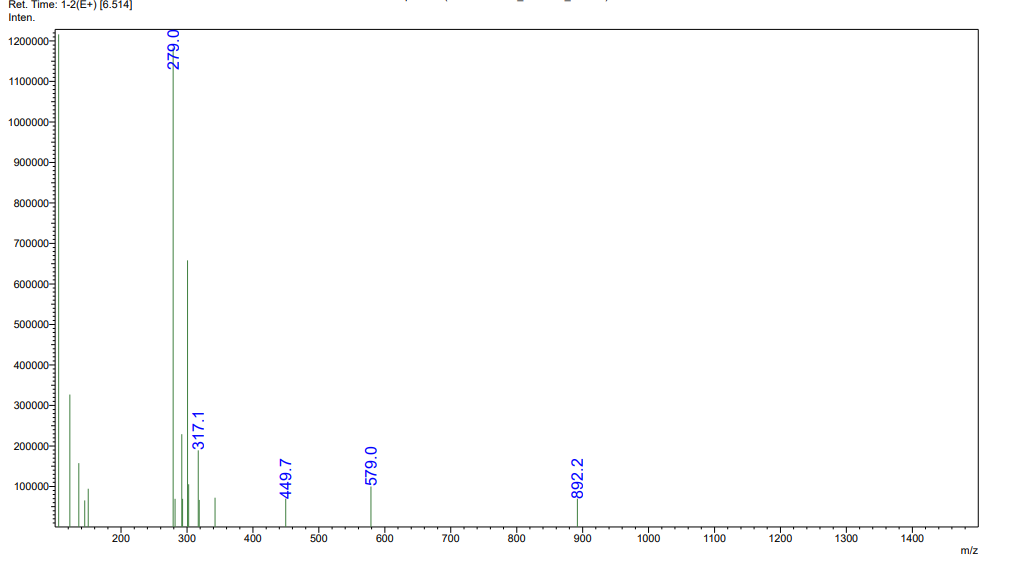
Supplementary Figure 11.** On-line mass spectrum of dihydrotanshinone I (**4**).

**Supplementary Figure 12.** On-line mass spectrum of danshenol A
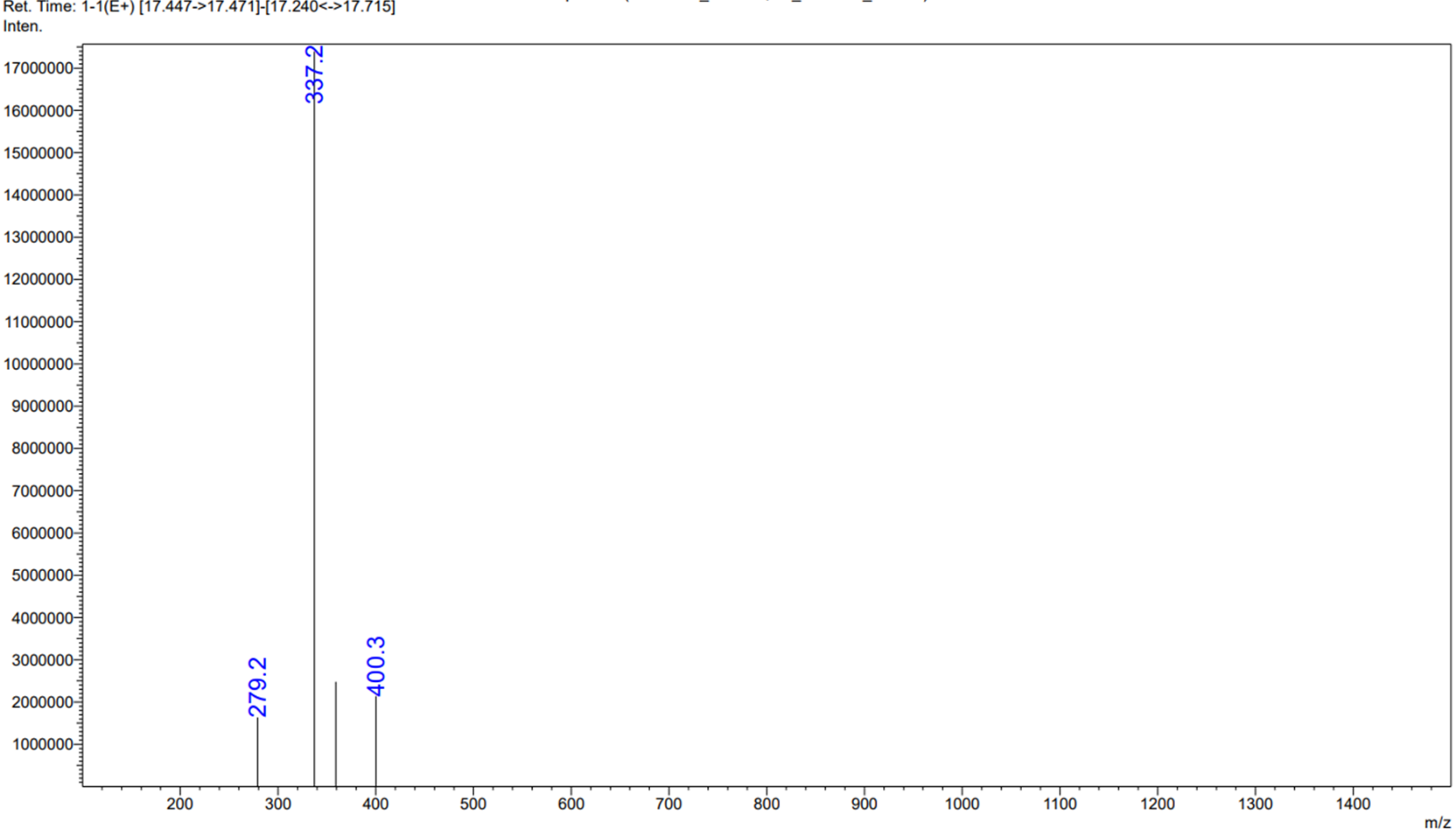
 (**5**).

**Supplementary Figure 13.** On-line mass spectrum of (+)-danshexinkun A
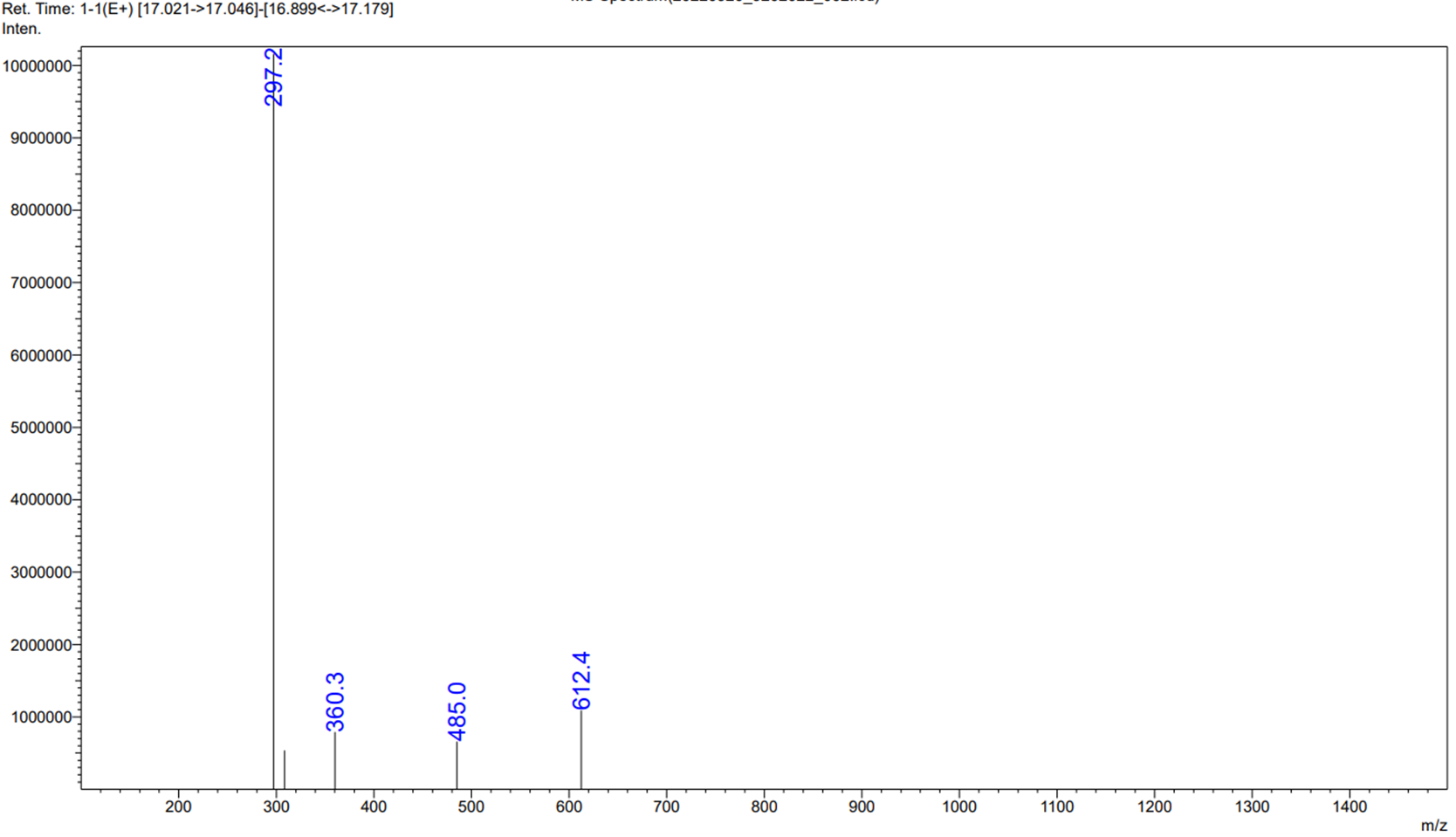
 (**6**).


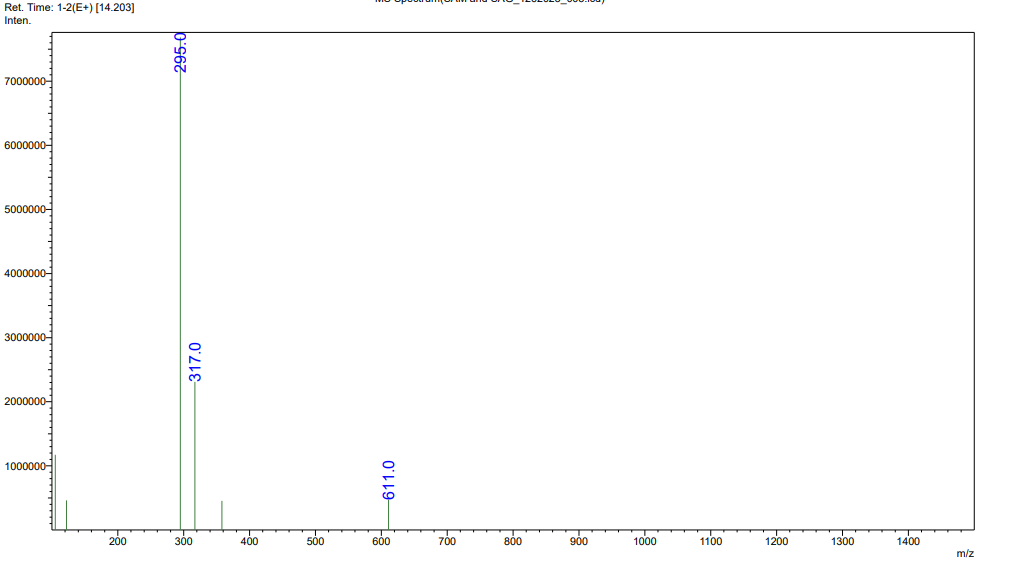


**Supplementary Figure 14.** On-line mass spectrum of tanshinone IIA (**7**).


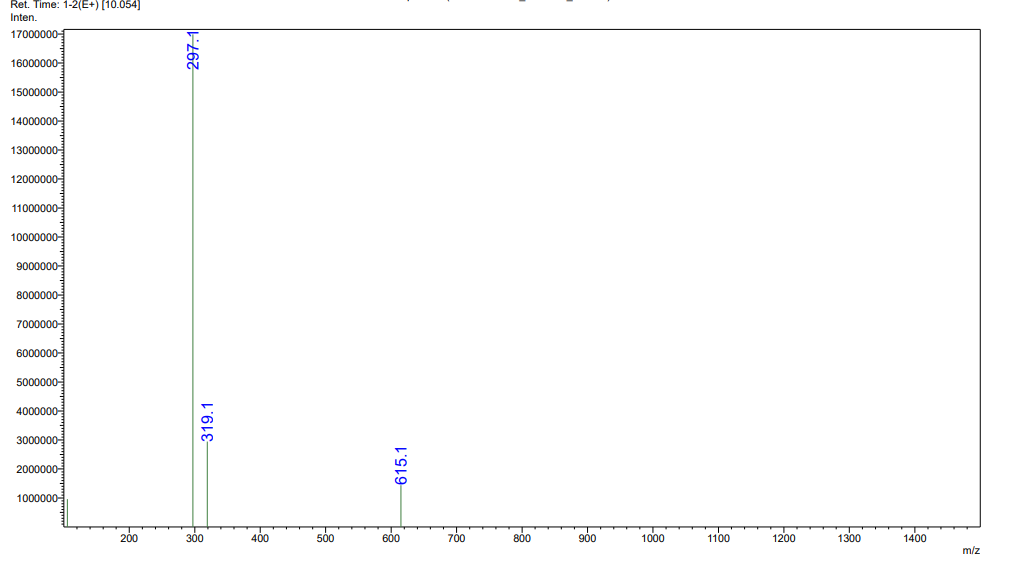


**Supplementary Figure 15.** On-line mass spectrum of cryptotanshinone.


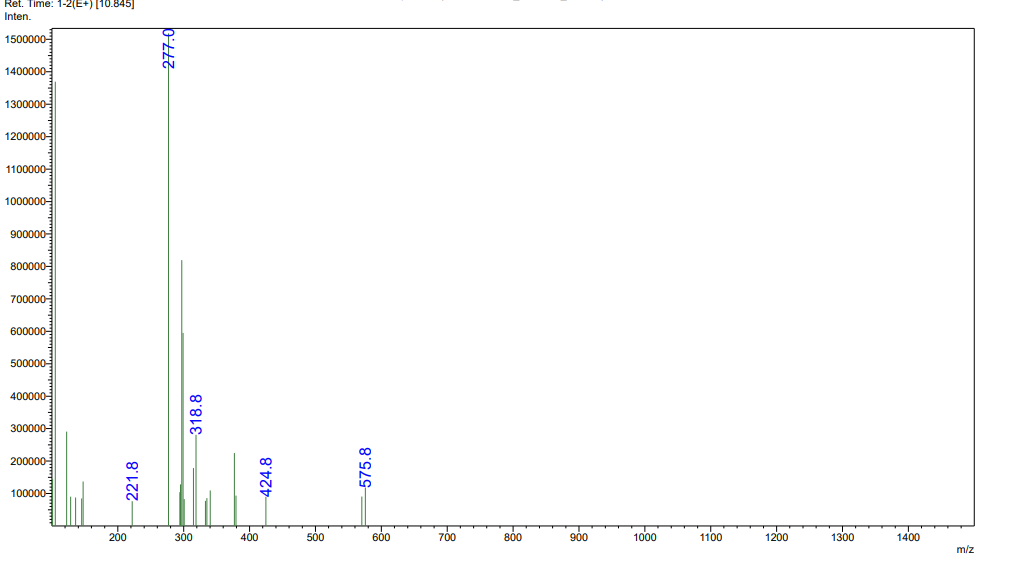


**Supplementary Figure 16.** On-line mass spectrum of tanshinone I.

**
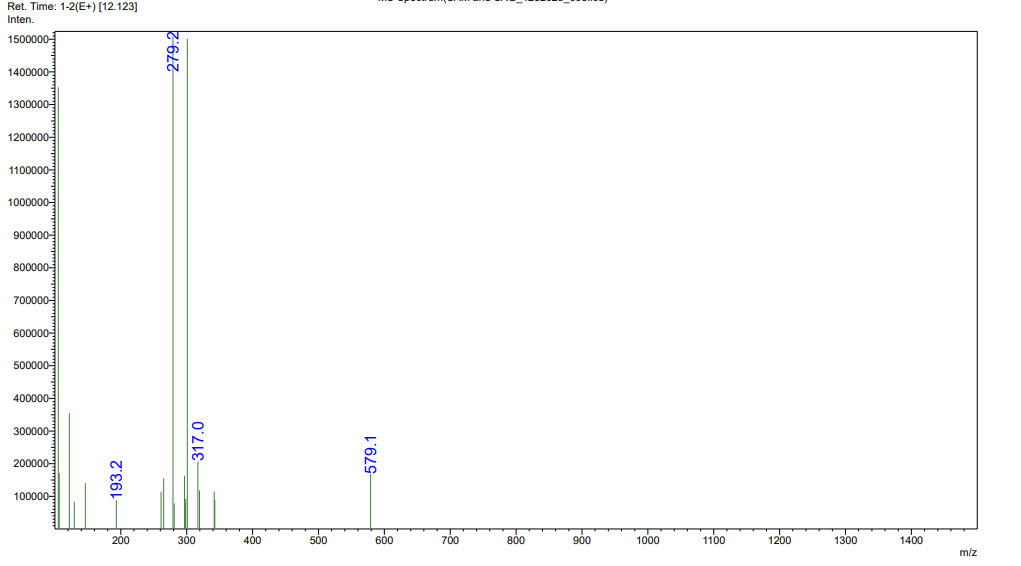
**

**Supplementary Figure 17.** On-line mass spectrum of 1,2-dihydrotanshinquinone/ methylenetanshinquinone.
